# Volume Fluctuations in Active and Nonactive Transtibial Prosthetics Users

**DOI:** 10.1155/2022/2669484

**Published:** 2022-09-12

**Authors:** Nur Afiqah Hamzah, Nasrul Anuar Abd Razak, Mohd Sayuti Ab Karim, Siti Zuliana Salleh

**Affiliations:** ^1^Department of Biomedical Engineering, Faculty of Engineering, University of Malaya, Kuala Lumpur, Malaysia; ^2^Department of Mechanical Engineering, Faculty of Engineering, University of Malaya, Kuala Lumpur, Malaysia

## Abstract

This study aims to evaluate the validity and reliability of the Biosculptor's Bioscanner system in capturing transtibial residual limb volume fluctuations in active and nonactive amputees during walking activity. Residual limb volume was obtained by measuring the limb circumference after amputees walked for 5 to 25 minutes for five consecutive days. The comparison of mean circumference between Bioscanner and manual measurements (i.e., tape measure) showed that the Bioscanner gave a higher estimation of circumference for the different amputees. Short-term changes in girth and volume due to an activity such as walking do not fluctuate uniformly. The results reflected as such as nonconsistence circumference change identified at different locations of the circumference profiles. Both amputees experienced a significant increase in circumference at the distal end of the limbs after 5 minutes of walking (7.35% change in nonactive and 8.83% in active amputees), and the measurement decreased as amputees walked longer. At 4-8 cm below the mid-patella tendon (pressure tolerant areas), both amputees experienced minor changes in the size of their circumference. The residual limb volume calculation resulted in the percentage difference between the two methods ranging from 2.4% to 9.3%. Pearson coefficient correlation obtained showed a high correlation between the two techniques, ranging from 0.97 to 1. The analysis of the limit of agreements showed that the majority of measurements were closed to the mean, suggesting that Bioscanner and manual techniques may be interchangeable and agree with one another. This study has implied that Bioscanner is comparable to the standard measurement method and may serve as an alternative tool in managing daily residual limb volume change.

## 1. Introduction

The prosthetic socket is a rigid and solid structure that has to work in unison with the dynamic body system that is constantly changing. Transtibial amputees managed the day-to-day shape and volume fluctuation of their residual limb by donning and doffing their prosthetic sockets and socks [1–3]. In prosthetic care, the changes in residual limb volume and shape are the sources of prosthetic socket fit issues. Small changes as small as 1% of residual limb volume crucially affect socket fit clinically [4]. Therefore, ineffective assessment and management of the residual limb can induce stress at the socket-limb interface, which leads to soft tissue damage and gait asymmetry [5, 6].

Transtibial amputees rely on the mechanical coupling between the socket and the residual limb to efficiently move daily. The residual limb is under continual stress especially during weight-bearing activities such as walking. However, the rate of daily limb fluid volume loss is different between amputees. Factors such as health level, limb-socket interface, and type of suspension influence the shape and fluid volume change of the amputees' residual limb [3, 7]. Age also influenced the level of ambulation in lower limb amputees [8].

The age factor highlighted earlier on the studies done by Durance et al. (1989) and Siriwardena and Bertrand (1991) [9, 10]. Meanwhile, healthy younger active transtibial amputees are expected to have the shape of the residual limb to be little to no change during simple daily activity [11]. The limb volume and shape do not fluctuate uniformly during certain activities. Areas of the limb may change a little, while others undergo dramatic shape change [12]. Additionally, according to Tyler et al. (2019), transtibial amputees experienced less fluid volume loss during a high activity compared to when they carried out a low-energy activity [3]. There is a direct correlation between the level of activity and the rate of residual limb volume loss. Due to this, prosthetists have to assess amputees' activity level before consulting them regarding limb volume management, and follow-up measurement is also necessary. This continuous measurement method is equally clinically critical to monitor shape and volume changes over time.

Therefore, the tool to manage residual limb volume needs to be reliable, nonexpensive, fast, and easy to use. The tool must accurately measure, capture, and differentiate the different rate of shape change based on the patient's level of ambulation and daily activities. [13–15]. By considering this, prosthetists can readily assist the patient in designing limb volume management specific for that patient based on the map of changes acquired from the measurement tool. Various novel methods and their measurement properties have been studied in residual limb shape management strategies, such as the water immersion method [16], ultrasound measurement [17, 18], computed tomography [19], and the computer-aided design and computer-aided manufacturing (CAD/CAM) laser scanning method [20, 21]. Sanders et al. (2012, 2018) also have comprehensively used bioimpedance analysis to investigate residual limb fluid volume change [2, 22].

Recently the CAD/CAM shape capturing scanning method has been the most successful alternative approach. It is applied not only in socket manufacturing but may also have the potential in managing rapid limb volume change. CAD/CAM systems such as CAPOD and Omega Tracer system are examined to be one of the most accurate systems [23]. Another recent study conducted by Mehmood et al. (2018) examined the potential of Biosculptor's Bioscanner system in measuring the circumference and volume of the transtibial residual limb. The study found that the Bioscanner measurements obtained were comparable to conventional socket [24].

Thus, this investigation aims to evaluate the reliability and validity of the Biosculptor's Bioscanner system in measuring residual limb reduction in real amputees after a daily activity. The study was designed to suggest Bioscanner as another alternative residual limb circumference measurement tool, by assessing the residual limb reduction/shape changes. The shape change is evaluated on active and nonactive unilateral amputees after subjects had walked [25], with the following hypotheses in consideration:

1. The rate of change in circumference is higher in active amputees
2. A long period of activity induces smaller shape changes in active amputees than the nonactive amputee
3. The Bioscanner can capture the daily changes on residual limbs comparable with the conventional method

## 2. Materials and Methods

### 2.1. Participant

The data was collected from two amputees recruited from the University Malaya Medical Centre (UMMC). This study was conducted with the approval and permission of the National Medical Research Register Secretariat 37912 and under the guidance and supervision of a certified prosthetist and orthotist (CPO) of the International Society of Prosthetist and Orthotics (ISPO) Category-2. Both amputees underwent thorough briefing sessions and were well-informed on the consent related to the study. The amputees were then categorized as nonactive and active amputees. The nonactive amputee was a female participant aged 68-year-old with a body mass index (BMI) of 33.3 kg/m^2^. The nonactive amputee was a unilateral transtibial (left) amputee with a history of amputation due to a diabetic ulcer in 2017 and was prescribed a patellar tendon-bearing socket (PTB) with an expanded polyethylene liner with a neoprene suspension sleeve. The active amputee was a 27-year-old male with a BMI of 17.4 kg/m^2^. The active amputee was also a unilateral transtibial (left) amputee with a history of amputation due to a traumatic traffic accident in 2013 that caused the lower limb to be amputated. The active amputee has been prescribed a PTB socket with an additional harness suspension.

The level of mobility of each subject was assigned according to the Medicare functional classification level. The active amputee was categorized as a K-4 level ambulator (high ability ambulation beyond the basic ambulation skills, typical prosthetic demand of an active ambulatory); meanwhile, the nonactive amputee was categorized as a K-2 level ambulator (limited community ambulator, with the ability to walk on level surfaces on fixed pace) [26]. Assessment of the participants' physical and prostheses characteristics is summarized in Table 1. The amputees' residual limb types and characteristics were determined by a prosthetist through limb palpation and visual examination. The residual limb shape of the active amputee was conical shaped and shorter and smaller than the nonactive amputee with a more cylindrical shape limb. The residual limbs were also checked to ensure that the skin condition was free of swelling, no phantom pain, sores, and skin ulceration.

Both amputees had to do a practice session to familiarize themselves with the laboratory condition. The practice session was crucial to detect any possible incidents that might occur during the data collection. The amputees were then instructed to carry out their typical daily routine and did not participate in any strenuous out-of-routine activities on the day of the experiment.

### 2.2. Experiment Protocol

**Figure 1:**
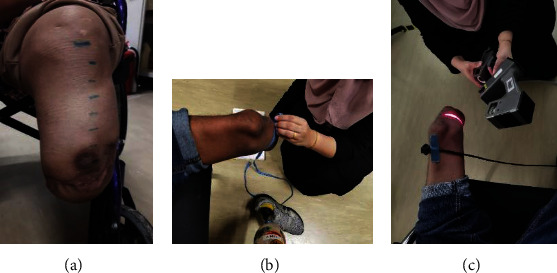
Circumferential intervals, started from the mid-patella tendon followed by 2-cm increment downward, the distal end was marked on participant's residual limb (a). A tape measurement (b) and the Bioscanner (c) were used to measure and capture the residual limbs. The digital images of the scanned limb were then directly displayed in FastScan software.

Both amputees were required to undergo a five day-long laboratory assessment. The subjects' residual limbs were measured before the walking exercise at 0 minutes. The measurements were obtained manually using a tape measure and digitally using the Bioscanner (FastSCAN, Polhemus). On the day of the assessment, the amputees were instructed to walk at their self-selected walking pace on a level floor. A set of measurements was obtained after 5, 10, 15, 20, and 25 minutes of walking, separated by two to five minutes of rest intervals. While resting, amputees were instructed to doff off their prostheses, and then the manual measurements using the measuring tape were first taken. Before measuring the residual limbs, the limbs had to be marked starting at the mid-patella tendon as point 0 cm. A certified prosthetist assisted in locating the mid-patella tendon and marked the residual limb accordingly. As shown in Figure 1, subsequent points are 2-cm increment downward towards the distal end of the residual limbs. The points made up the circumferential profiles of the residual limbs. Alternatively, the digital measurements were obtained using the Bioscanner system. It is an electromagnetic, noncontact scanning device that allows users to capture the residual limbs by sweeping the handheld device over the limb at a 45° angle. Bioscanner has a built in dual-camera system with a line laser equipped with a transmitter [27].

During the experiment, amputees' residual limbs were scanned without the socks/stockinet, which might obstruct the scanning process. The stylus mode of the laser was used to mark the mid-patella tendon. The scanning process also had to be done in a dimmed or darkened room to ensure no other light source would affect the accuracy of the device. While sitting down, a transmitter device was attached to the amputees' residual limb. The transmitter was attached to the patient acted as guide or “tracker” connected to the motion-tracking device integrated in the Bioscanner. The transmitter assisted in tracking each sweep profile and also stabilize the limb from any movement from the subject [28]. Posture or movement of the limb during flexion would not have a significant effect on the measurement. The “sweeping” started 2 cm above the mid-patella tendon point and ended at the distal end of the residual limbs.

Similar with most 3D scanner, the Bioscanner relied on geometry of light structures projected on the object in a form of multiple light stripes/lines (the triangulation principle) which is based on its own light source and detector (transmitter). Once the transmitter was placed at known distance, it was detected by the scanner as its electromagnetic reference. The Bioscanner can scan at a scanning rate of 50 lines per second with the accuracy of 0.75 mm. Subsequently, each sweep profile was uploaded directly into FastScan software. Five to eight sweeps (i.e., multiple images of parts of the residual limb) made up the full 3D impression of the residual limbs, and it was achieved in less than 5 minutes. Once the scans were completed, 3D impression of the residual limbs was then exported to CAD software called Bioshape. Bioshape allowed processes such as modification and rectification to perform digitally. Once the residual limbs were scanned, the software measured the circumferential profiles automatically.

For this study, the 3D images of the residual limbs were left unmodified. The circumferential profiles were obtained digitally by marking the images at 2-cm intervals starting at the mid-patella tendon as the most proximal point (0 cm). The mid-patella tendon landmarks were assigned using the optical stylus mode of the Bioscanner. Therefore, it is crucial to ensure the region of the mid-patella was marked correctly to ensure the circumferential profiles obtained digitally were comparable with the manual measurement.

### 2.3. Measurement Data Analysis

The data were collected and recorded for five consecutive days. After both participants completed the exercises, all results were analyzed based on the proposed hypotheses. The frustum model method was used to estimate the residual limb volume. The circumference values were incorporated using the following Equation (1). The formula for a paraboloid (1/2*πr*_*z*_^2^*h*_*z*_) was used to calculate the distal end of the limbs. This calculation was also described by Bolt et al. (2010) as a better approximation of the real shape of the residual limb [23].

(1) $$\begin{array}{c} V=\frac{\pi }{12\pi^{2}}\times h\left[\left(C^{2}+Cc+c^{2}\right)\right]+\frac{1}{2}\pi r_{z}^{2}h_{z}, \end{array}$$

where *C* is the uppermost disc of the residual limb (cm), *c* is the lowermost disc of the residual limb (cm), *h* is the total length of the residual limb/paraboloid, *V* is the volume of the residual limb, and *r* is the radius of paraboloid (calculated using the formula *r* = *c*/2*π*) with (*z*) as the most distal measurement.

The percentage of change of the residual limb against the time walked by amputees and the different between the two methods are calculated using the following equation:

(2) $$\begin{array}{c} \text{Percentage of differences }\left(\%\right)=\frac{\text{Difference of the }2\text{ values}}{\left(\text{Sum of the }2\text{ values}/2\right)}\times 100=\%. \end{array}$$

The data were analyzed using the Pearson correlation coefficients. The calculation allowed researchers to evaluate and validate the analogous relationship between the Bioscanner and the conventional tape measure method. Moreover, to evaluate whether the results obtained agreed with one another, graphical studies of the limits of the agreement were also conducted.

## 3. Results

**Figure 2:**
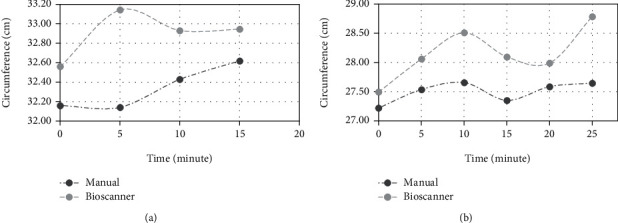
Changes in residual limb circumference (mean *n* = 5) at the different time interval during the walking activity for nonactive (a) and active amputee (b).

The active amputee managed to complete the five days of the experiment as planned without any health-related issues. In contrast, the nonactive amputee, due to the amputee's medical and physical conditions, the amputee only managed to walk for 0 to 15 minutes for the duration of five days [29]. Significant differences in the circumferential profiles of the amputees were found for both techniques. Overall, the Bioscanner estimates a slightly higher circumference over time than the tape measure, as illustrated in Figure 2. The analysis of the results displayed the highest mean difference in circumference in the nonactive amputee at 5 minutes (Figure 2(a)). The lowest difference was at 0 minutes with an approximate 0.27 cm difference in mean circumference between the active amputee's manual and Bioscanner measurement (Figure 2(b)). The active amputees recorded the highest deviation in mean at 25-minute intervals with only approximately a 1.14 cm difference. The circumference profiles in nonactive amputees were increasing over time in a nonlinear way. A similar result was observed for the active amputee's Bioscanner measurement, while the manual method measurement showed a change in the circumference approximated to linearity as the amputee walked longer.

**Figure 3:**
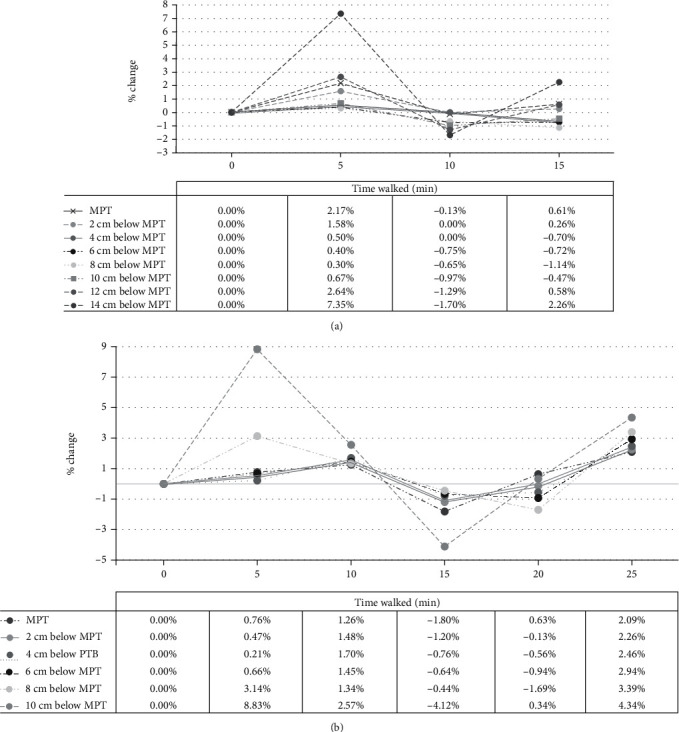
Percentage of change of the circumference against the time amputees walked measured by Bioscanner. Each line represented the measurement location of the circumferential profiles from 0 cm (MPT: mid-patella tendon) towards the subsequent 2-cm intervals for both nonactive (a) and active amputees (b).

As previously reported, short-term changes in the girth and volume of the residual limb due to activity such as walking do not fluctuate uniformly. To emphasize the differences in the shape changes at different locations along the limb, the percentages of circumference variation while amputees walked at different periods are displayed in Figure 3. Generally, both amputees resulted in a similar pattern over time. Both amputees experienced a significant increase at 14 cm below MPT for nonactive and 8.83% increased at 10 cm below MPT for active. However, as amputees walked longer, the circumference of the distal end decreased rapidly. In the nonactive amputee, the measurement of the circumference of the distal end went from 27.51 cm to 27.05 cm, a 1.7% difference, after 5 minutes, while the active amputee went from 24.16 cm to 23.76 cm, a decline of 2% after 15 minutes of walking. Approximately 4-8 cm below the MPT, where the pressure tolerant areas were, both amputees experienced minor changes in the size of their circumference (ranging from 0%-1.14% in nonactive to 0.21%-2.9% in the active amputee) [30]. The figure also presented that the active amputee's pressure-tolerant areas were more stable with <4% change throughout the activity.

**Figure 4:**
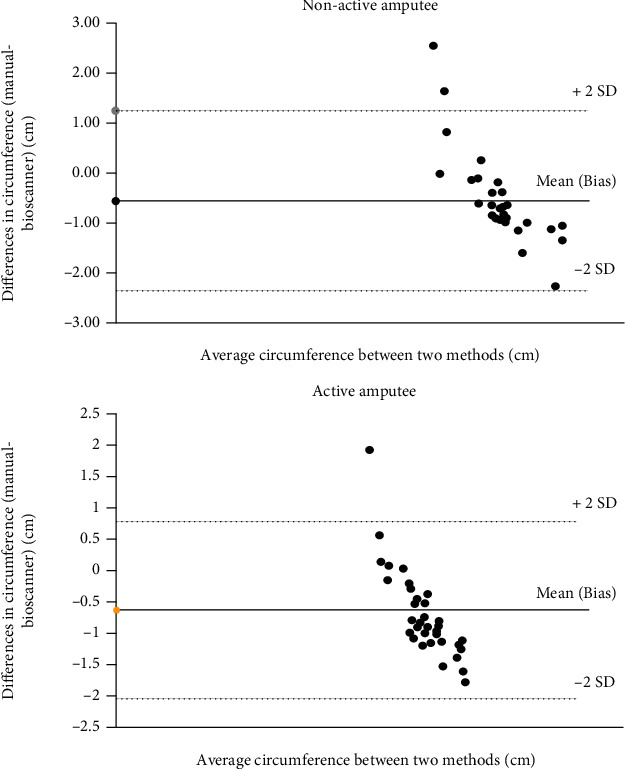
Limits of agreement of the manual tape measure measurement and the Bioscanner method.

To measure the reliability of the Bioscanner in capturing the daily changes of the residual limb, the circumference measurements were used to calculate the volume of the residual limb that occurred during the observation and compared with the manual measurements. The data indicated in Table 2 showed that at 0 minutes, the percentage differences were the lowest for the two methods, while the highest recorded volume for active amputees was at 25 minutes and at 5 minutes for the nonactive amputee. The results also showed that the average percentage difference between residual limb volumes of nonactive amputees was less than 3%. In addition, the study managed to obtain an excellent correlation coefficient between the methods ranging from 0.991 to 1.0. The study on the limit of agreements allows the study to determine whether the two techniques can be interchangeable and agree with one another as according to Figure 4. Most of the measured data were closed to the mean, and only 3 data were classified as outliers.

## 4. Discussions

Data on the circumferential profiles allowed prosthetists to design and assist in the prosthetic socket and residual limb shape management. In this study, Bioscanner was proposed as a method to measure the changes in the circumference of amputees' residual limb after walking. Comparison of the mean circumferences between the manual method and Bioscanner, as illustrated in Figure 2, shows that Bioscanner consistently overestimated the measurement than the manual method for both amputees. The underestimation in manual measurement may be due to the limb muscles being pressed slightly during measurement taking, while the Bioscanner involved no contact with the surface of the limb [20]. The difference between the two techniques is less than 10% (Table 2). The highest differences occur at the distal end of the limbs for both amputees. The characteristics of the distal end of the residual limb, which is less bony, contributed to the significant difference in measurement.

The effort in mapping the nonuniform changes along the residual limb also provided the data for designing residual limb volume control strategies. The study showed that Bioscanner had the potential to capture the residual limb shape change after walking. As the session progressed and both amputees walked longer, the shape of the residual limb stabilized. Based on Figure 2, the active amputee experiences a higher rate of change during the activity when compared to the nonactive amputee, which validated the first hypothesis. Both amputees' distal ends of the limb underwent an extreme volume fluctuation due to the “unrecoverable” soft tissue situated in this area. Unrecoverable soft tissue will experience deformities and loses fluid volume when load and stress are applied [31]. A similar report was presented by Youngblood et al. (2019), where the study emphasized that an active amputee tends to expect high fluid volume loss at the start and less loss lesser later when carrying out an activity [3]. An earlier study conducted by Zachariah et al. (2004) also showed that the distal half of the residual limb indicated a higher volume increase during a short-term activity and the rate of volume decreased with time [32]. Based on the circumference percentage difference in this study, both active and nonactive amputees induced less shape fluctuation after a long period of activity, which tallies with the second hypothesis. However, the walking pace between the two amputees may also need to be considered, as a nonactive amputee coupled with old age, comorbidities, and lack of physical strength tend to walk slower, thus producing a lesser variation in circumference measurement along the residual limb [33]. The analysis of the percentage of shape changes between the active and nonactive amputees using the Bioscanner showed that it is a reliable method to use.

According to a study conducted by Johnson et al. (1995), lower limb amputees with medical problems, mainly chronic obstructive pulmonary disease (COPD) and peripheral vascular disease (PVD) and diabetes mellitus, scored both the pre-and post-amputation mobility scores the lowest [34, 35]. The importance of the effect of these comorbidities during the evaluation of the amputee's potential to use a prosthesis was observed. Nonetheless, based on the study, nonactive amputees acquired a similar rate of circumference change pattern to the active amputee. This is indicative of successful rehabilitation and pre-post prosthetic training on the nonactive amputee. Additionally, a study done by Sanders et al. (2014) indicated that walking with the prosthesis in transtibial amputees only accounted for 39.3% of total fluid volume change [36]. The Bioscanner system showed high consistency and strong correlation with the conventional tape measure; thus, the data is valuable for prosthetists to consider routinely using Bioscanner to capture daily residual limb changes. The limits of agreement analysis also provide the user that Bioscanner may justify that the method is reliable and interchangeable with the conventional tape measure method.

## 5. Conclusion

The assessed results indicate that Bioscanner is an appropriate method of fast capturing the shape of residual limb. The data presented in this study provides important information regarding the rate of circumference change in transtibial amputees based on their ambulation level. The system can used in the stage of pre-post prosthetic training of lower limb amputee due to its feasibility to detect the variations in circumference along the residual limb in active and nonactive amputees. The high coefficient correlation presented also showed that Bioscanner may be a reliable complementary measurement tool in measuring daily changes in residual limb. As the study was done and conducted with human subjects, issues such as tiredness and health issues contributed to the rate of circumference change of the participants' residual limb [37]. Although the study has compared between two different levels of ambulators, the contribution of factors such as walking speed, the large age gap, and participants' diet may also affect the results. The study also did not fully examine the capability of the system in the modification stage for both amputees. In addition, further investigation can be conducted on larger set of participants to demonstrate greater clinical impact of the Bioscanner as a measuring tool for limb shape management.

## Figures and Tables

**Table 1:** Participants' details and prostheses characteristics.

Amputee	Gender	Age (year)	BMI	Time since amputation (year)	Amputation etiology	Limb shape	Amputation side	Residual limb length (cm)	Socket design	Activity level
1	Female	68	33	2	Vascular disease	Cylindrical	Left	17.4	PTB	K-3
2	Male	27	18	7	Trauma	Conical	Left	12.5	PTB	K-4

PTB: patella tendon bearing; BMI: body mass index.

**Table 2:** Comparison of manual with Bioscanner method on the estimated residual limb volume for the average of 5 days and its percentage differences for active and nonactive amputees.

Active	Walking period (minutes)					
	0	5	10	15	20	25
Manual (ml)/SD	877.76/0.7	901.83/0.4	901.18/0.4	884.25/0.3	903.12/0.4	904.92/0.4
Bioscanner (ml)/SD	908.95/1.7	948.06/0.6	973.75/0.8	947.89/0.8	940.71/0.9	993.56/0.8
% differences	3.49	5.00	7.74	6.95	4.08	9.34
Correlation coefficient	0.99	1.00	1.00	0.99	1.00	1.00

Nonactive	Walking period (minutes)					
	0	5	10	15		
Manual (ml)/SD	1772.68/0.6	1768.11/0.6	1839.70/0.6	1863.30/0.6		
Bioscanner (ml)/SD	1822.62/0.5	1915.80/0.3	1890.70/0.4	1908.69/0.3		
% Differences	2.78	8.02	2.73	2.41		
Correlation coefficient	0.97	1.00	0.98	1.00		

SD: standard deviation.

## Data Availability

The data used to support the results of this study are available from the corresponding author upon request.
